# Childhood obesity from the genes to the epigenome

**DOI:** 10.3389/fendo.2024.1393250

**Published:** 2024-07-09

**Authors:** Senthil Sivakumar, Dechen Lama, Nabil Rabhi

**Affiliations:** Department of Biochemistry and Cell Biology, Boston University Chobanian & Avedisian School of Medicine, Boston, MA, United States

**Keywords:** obesity, maternal obesity, paternal obesity, transgenerational, intergenerational, epigenetics

## Abstract

The prevalence of obesity and its associated comorbidities has surged dramatically in recent decades. Especially concerning is the increased rate of childhood obesity, resulting in diseases traditionally associated only with adulthood. While obesity fundamentally arises from energy imbalance, emerging evidence over the past decade has revealed the involvement of additional factors. Epidemiological and murine studies have provided extensive evidence linking parental obesity to increased offspring weight and subsequent cardiometabolic complications in adulthood. Offspring exposed to an obese environment during conception, pregnancy, and/or lactation often exhibit increased body weight and long-term metabolic health issues, suggesting a transgenerational inheritance of disease susceptibility through epigenetic mechanisms rather than solely classic genetic mutations. In this review, we explore the current understanding of the mechanisms mediating transgenerational and intergenerational transmission of obesity. We delve into recent findings regarding both paternal and maternal obesity, shedding light on the underlying mechanisms and potential sex differences in offspring outcomes. A deeper understanding of the mechanisms behind obesity inheritance holds promise for enhancing clinical management strategies in offspring and breaking the cycle of increased metabolic risk across generations.

## Introduction

The global rise in obesity presents a pressing public health challenge that demands thorough exploration to understand its complex origins and extensive impacts. According to the World Health Organization (WHO) in 2016, a staggering 39% of adults aged 18 years and over were classified as overweight, amounting to 1.9 billion individuals, with an alarming 13% falling into the obese category (World Health Organization, 2016). Projections by the World Obesity Federation suggest that if current trends persist, more than half (51%) of the global population will be affected by overweight or obesity by 2035 (1). Overweight and obesity are distinguished by Body Mass Index (BMI), with a BMI of ≥ 25 kg/m² indicating overweight and ≥ 30 kg/m² indicating obesity (WHO). This epidemiological trend carries significant implications, as obesity is closely linked to a myriad of health conditions, including cardiovascular disease (CVD), gastrointestinal disorders, type 2 diabetes (T2D), musculoskeletal disorders, respiratory complications, nonalcoholic fatty liver disease (NAFLD), chronic kidney disease (CKD), cancer, and psychiatric disorders (1).

The childhood obesity epidemic is equally worrisome, demanding urgent attention. In 2020, an alarming 39 million children below the age of 5 were affected by overweight or obesity, while over 340 million children and adolescents aged 5-19 faced similar challenges in 2016 (World Health Organization). What’s particularly concerning is the steady rise in the age-standardized mean BMI of children aged 5 to 18, which has increased globally by 0.32 kg/m² per decade from 1975 to 2016 . The consequences of childhood obesity are profound, encompassing an increased risk of lifelong obesity, the onset of chronic illnesses, and various psychosocial impacts (2–7). Notably, the challenges posed by the COVID-19 pandemic, including lockdowns and disruptions to routines, have further exacerbated the issue, leading to a troubling surge in childhood obesity rates (8). At its core, obesity arises from a complex interplay of imbalanced energy intake and expenditure, influenced by various factors such as changing lifestyles and dietary patterns (9, 10). Total energy expenditure includes basal metabolic rate, energy expended at rest, and physical activity expenditure. Recent trends underscore a societal transition toward a more sedentary way of life, contributing to diminished energy expenditure. Additionally, the obesogenic environment, characterized by easy access to high-calorie foods and limited physical activity opportunities, has fueled the prevalence of obesity. However, over the past decade, it has become clear that additional factors are involved (11).

Indeed, analysis of dietary data from the National Health and Nutrition Survey (NHANES) during the period from 1998 to 2006 found a noteworthy average increase of 2.3 kg/m² in BMI within the USA, even when accounting for dietary intake and exercise levels (12). These findings imply that factors beyond dietary habits and exercise participation warrant thorough investigation. Several plausible etiological factors for obesity have been posited, encompassing chronic sleep deprivation and circadian misalignment (13), alterations in the gut microbiome (14), and specific pharmaceutical agents known to induce weight gain (11, 15). Additionally, a multitude of genome-wide association studies (GWAS) have been conducted to elucidate genetically mediated heightened susceptibility to obesity (16). It is noteworthy, however, that genetic variants associated with obesity exhibit limited predictive capacity, collectively accounting for a mere ~3% of the variance in BMI (17). Finally, several lines of evidence demonstrated that environmental factors and alterations in nutritional conditions *in-utero* can exert direct influences on the epigenome of ancestral germline cells consequently imparting susceptibility to obesity (18–23). This mechanism is recognized as transgenerational or intergenerational inheritance of obesity. Notably, a recent meta-analyses, including 23 clinical studies, indicate a significant link between parental obesity and childhood obesity, showing that children with overweight or obese parents are nearly twice as likely to be obese compared to those with normal-weight parents (24). The risk escalates considerably when both parents are obese (odds ratio: 12.0), and even more so when both parents are severely obese (odds ratio: 22.3), independent of age, gender, socioeconomic status, and ethnicity (25). Moreover, studies predicting the risk of childhood or adolescent obesity have found that parental BMI and socioeconomic factors are better predictors of childhood obesity than genetic scores (26, 27). While both maternal and paternal obesity significantly contribute to the risk of childhood obesity, findings regarding the relative contributions of each parent vary across studies. Some studies suggest that maternal obesity has a stronger impact (25, 28, 29), while others indicate a greater influence from paternal obesity (26, 30, 31), highlighting the complexity and the need for further research to fully understand these dynamics.

In this review, we delve into the current evidence and molecular mechanisms underlying the inheritance of obesity, with a particular emphasis on inter- and trans-generational transmission. To ensure a thorough exploration of the topic, we conducted a comprehensive literature search, meticulously gathering relevant studies that shed light on the inheritance patterns of obesity. Our search encompassed a wide range of sources, including data on familial clustering of obesity, genetic studies elucidating predisposing factors, and animal models providing insights into transgenerational inheritance phenomena. The criteria for selecting studies were rigorously defined, prioritizing relevance to inter- and trans-generational inheritance and ensuring the robustness of review designs employed. By adopting this strategic approach, we aimed to provide a comprehensive overview of the inheritance mechanisms contributing to the obesity epidemic, thereby advancing our understanding of this complex and pressing public health issue.

**Figure 1:**
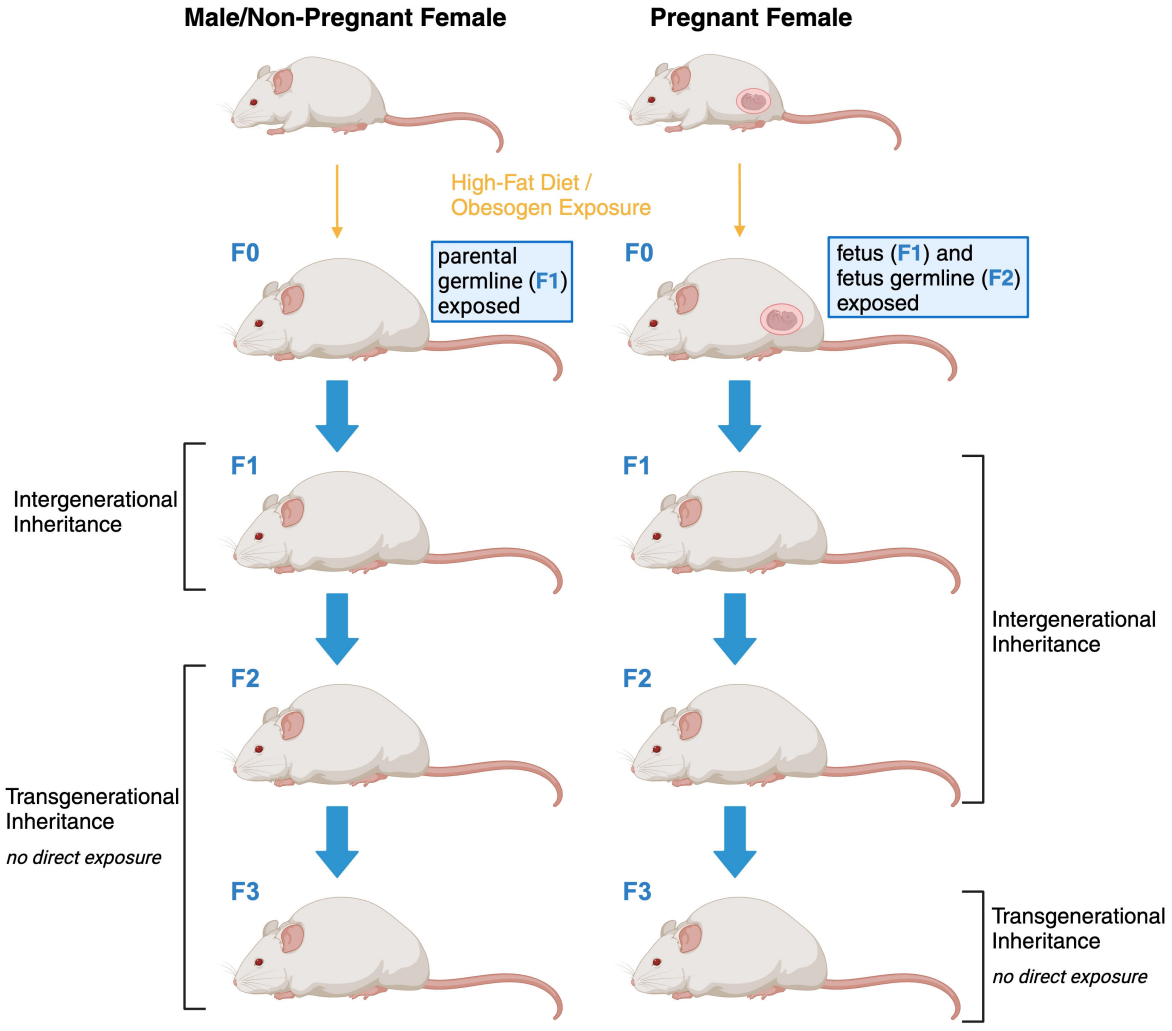
Schematic representation of intergenerational and transgenerational inheritance of obesity.

The terms transgenerational and intergenerational are often used interchangeably when obesity-related effects are discussed hence before we continue it is opportune to clarify the definitions as previously established and used in this manuscript (**Figure 1**). The notion of ‘transgenerational effects’ encompasses phenomena exclusively attributed to factors that cannot be ascribed to the direct impact of a particular trigger on the affected organism. For instance, an environmental stimulus can directly influence a developing embryo, including the already-formed oocytes within a female embryo in mammals (32–34). Consequently, only modified phenotypes that emerge in the second generation (in the case of male transmission) or the third generation (in the case of female transmission) subsequent to a trigger can be accurately characterized as transgenerational inheritance in the context of obesity. In contrast, effects manifesting over shorter temporal intervals are categorized as ‘parental’ or ‘intergenerational’ in nature. It is important to note that while these intergenerational effects span fewer generations, they may share underlying mechanistic pathways with transgenerational effects.

## The genetics of obesity

The heritability of obesity is estimated to range from between 40% and 75% (35). Genetic forms of obesity manifest along a continuum of clinical features traditionally classified into three overarching categories: Mendelian (monogenic) syndromic obesity, Mendelian non-syndromic obesity, and polygenic obesity (36).

Mendelian obesity forms result from rare chromosomal abnormalities and pathogenic gene variants that impact critical proteins involved in regulating energy balance. They are typically rare, early-onset and adhere to a Mendelian inheritance pattern and can be either autosomal or X-linked (37, 38). Syndromic forms of Mendelian obesity, also referred to as pleiotropic syndromes, are relatively uncommon within the general population. Syndromic obesity is characterized by obesity alongside additional distinctive features such as intellectual disabilities, dysmorphic traits, and congenital anomalies affecting specific organ systems. About 79 syndromes have been associated with obesity; notable examples include Albright hereditary osteodystrophy, Alström, Bardet-Biedl, and Prader-Willi syndrome (39). Those have been reviewed recently by Kaur et al. and will not be further discussed here (39).

**Figure 2:**
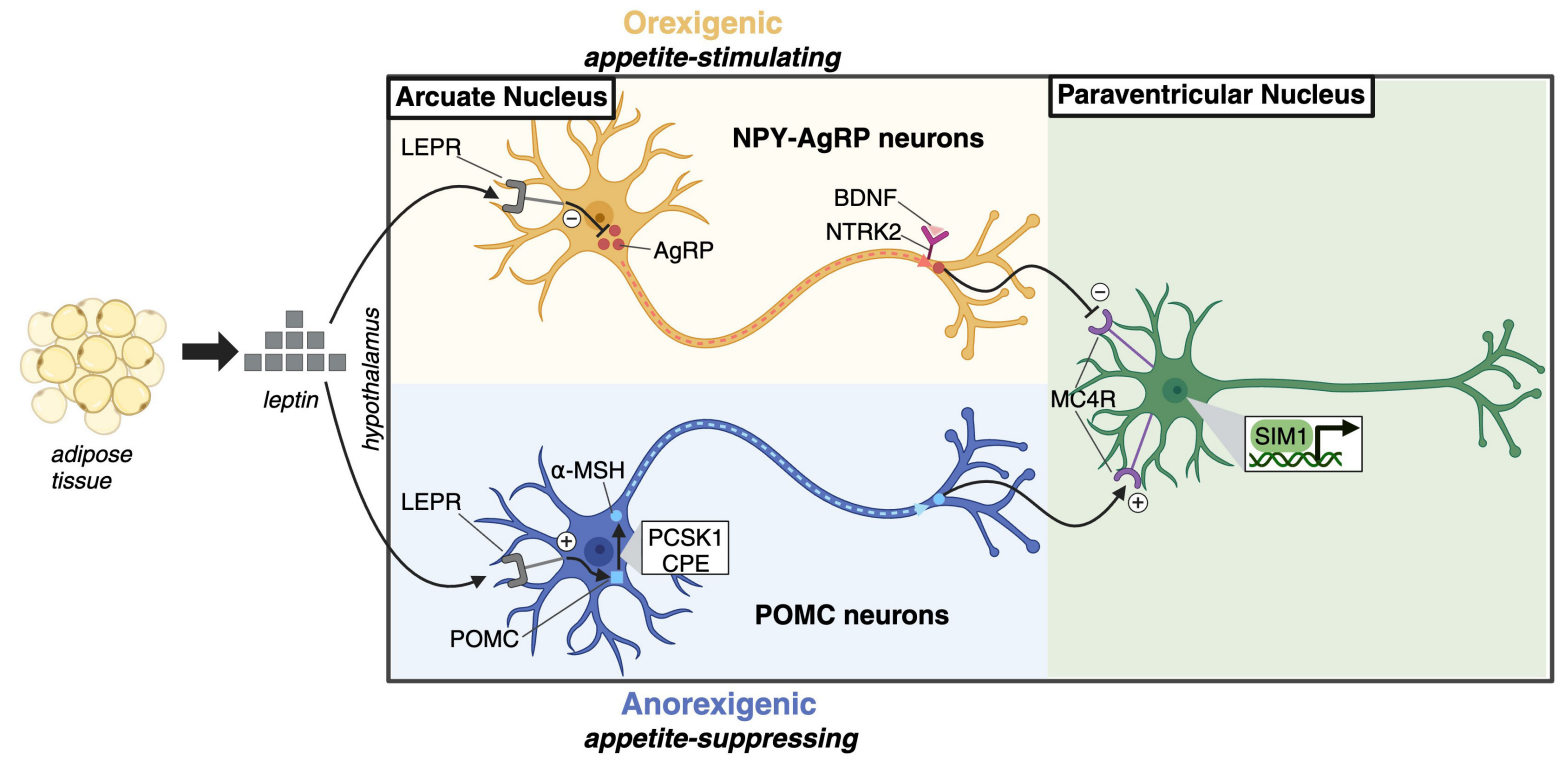
Leptin-melanocortin pathway. Leptin, primarily secreted by adipocytes, acts as a satiety factor in the hypothalamic arcuate nucleus. It binds to the leptin receptor (LEPR) on two neuron populations: neuropeptide Y (NPY)–agouti-related protein (AgRP) neurons and pro-opiomelanocortin (POMC) neurons. This binding downregulates orexigenic AgRP production in NPY-AgRP neurons and upregulates anorexigenic α-melanocyte-stimulating hormone (α-MSH) in POMC neurons. α-MSH acts as an agonist, while AgRP serves as a reverse agonist of MC4R on neurons in the paraventricular nucleus, signaling satiety and limiting food intake. Brain-derived neurotrophic factor (BDNF) modulates leptin-mediated synaptic plasticity through its receptor neurotrophic receptor tyrosine kinase 2 (NTRK2), while Single-minded homologue 1 (SIM1) is a transcription factor essential for the development of paraventricular nucleus neurons.

On the other hand, non-syndromic Mendelian obesity forms identified thus far are primarily associated with genetic defects in the leptin/melanocortin pathway, leading to hyperphagic obesity (40, 41). These encompass mutations in genes encoding key components such as leptin, the leptin receptor, prohormone convertase 1, pro-opiomelanocortin, or melanocortin 4 receptor (**Figure 2**).

In contrast to monogenic obesity, polygenic obesity does not stem from a single gene with a significant impact on obesity development. Polygenic obesity is believed to be determined by the cumulative influence of numerous common genetic variants, each exerting modest effect. For instance, common variants located within intron 1 of the fat mass and obesity-associated gene (FTO) are among the most prominent contributors to polygenic obesity, accounting for approximately 1% of the variance in BMI within the general population.

Polygenic obesity, commonly known as common obesity, differs markedly from monogenic obesity, as it does not arise from a singular gene exerting a substantial impact on obesity development. Instead, polygenic obesity is shaped by the collective influence of numerous common genetic variants, each exerting modest effects (42–44). For example, common variants located within intron 1 of the fat mass and obesity-associated gene (FTO) are among the most prominent contributors to polygenic obesity, while accounting for only 1% of the variance in BMI within the general population (43, 44). This multifactorial condition aligns with heritability patterns observed in complex traits and diseases and shares a foundational biological framework with monogenic obesity. Specifically, both forms of obesity implicate the central nervous system (CNS) and the neural pathways governing food intake’s pleasurable aspects as crucial determinants of body weight regulation (45). Furthermore, emerging evidence suggests that mutations responsible for monogenic obesity may, to some extent, be influenced by an individual’s polygenic predisposition to obesity (46).

Genome-wide association studies (GWAS) have accelerated the identification of loci linked to polygenic obesity. Obesity-associated gene (FTO) was the first GWAS-identified obesity gene. The FTO locus has a well-established correlation with obesity but the specific mechanisms connecting FTO polymorphisms to obesity risks still need to be researched. Several loci discovered by GWAS are near genes that are also associated with monogenic obesity, including MC4R, BDNF, SH2B1, POMC, LEP, LEPR, NPY, SIM1, NTRK2, PCSK1 and KSR2 (45) (**Table 1**).

**Table 1:** List of genes found to control obesity inheritance in mice models and their functions.

Gene	Function	Mutant Phenotype	References
LEP	Signals satiety and binds to neurons in the hypothalamus	Hyperphagia, early-onset obesity, hypogonadotrophic hypogonadism, weakened immunity	(47, 48)
LEPR	Leptin-receptor present on 2 different neuron populations (NPY-AgRP and POMC) in the arcuate nucleus of the hypothalamus	Hyperphagia, early-onset obesity, hypogonadotrophic hypogonadism, weakened immunity	(49, 50)
MC4R	G-Protein coupled receptor present on neurons in the hypothalamus that regulates satiety	Hyperphagia, early-onset obesity	(51, 52)
POMC	Cleaved to form melanocortin peptides like the MC4R agonist α-MSH	Hyperphagia, early-onset obesity, hypoadrenalism, red hair and pale skin	(53)
PCSK1	Catalyzes the cleavage of the POMC protein	Early-onset obesity, hypogonadotrophic hypogonadism, hypocortisolism, elevated plasma proinsulin and POMC concentrations, very low insulin levels, abnormal glucose homeostasis	(54)
SIM1	Transcription factor necessary for the development of neurons of the paraventricular nucleus	Hyperphagia, reduced energy expenditure, obesity	(55–57)
SEMA3A-G	Direct the development of GnRH neurons in the hypothalamus	Obesity, hypogonadotropic hypogonadism	(58)
BDNF	Modulates synaptic plasticity of neurons	Hyperphagia, severe obesity, and impaired cognitive function	(59–61)
NTRK2	Receptor of BDNF	Hyperphagia, severe obesity, and impaired cognitive function	(62, 63)
IGF2	**Imprinted gene**: Establishes insulin resistance state in mother, regulates nutrient transport to fetus	Prader-Willi Syndrome: Hyperphagia, early-onset obesity, hypotonia, growth retardation, altered intellectual and sexual development, sleep disturbances	(64)
Pref1/Dlk1	**Imprinted gene**: Inhibits adipogenesis, regulates metabolism and development	Obesity, growth retardation, blepharophimosis, skeletal malformation, and increased serum lipid metabolites	(65)
Peg1/Mest	**Imprinted gene**: *Exact function unknown*	Increased body weight and increased weight of kidney and spleen *(in mice)*	(66, 67)
TRIM28	Epigenetic modifier complex mediated by a cohort of KRAB Zinc finger proteins	Bi-stable obesity	(68)
Spag7	Embryonic development, energy homeostasis	Obesity, insulin-resistance, low fetal birthweight, reduced skeletal muscle activity *(in mice)*	(69)

## Mechanisms for inter/transgenerational inheritance

Intergenerational phenotypes encompass alterations that arise from changes in ancestral generations’ phenotypes, which are distinct from primary DNA sequence modifications. These alterations involve various epigenetic modifications, including DNA methylation, histone modifications, and non-coding RNAs. The epigenome is remarkably sensitive to environmental influences such as lifestyle, dietary patterns, gut microbiota, and other factors (70, 71). Exposure early in life or *in utero* to those can alter metabolic outcomes through developmental epigenetic reprogramming (72). Understanding the mechanisms underlying intergenerational inheritance remains a complex challenge, as maternal lineage is influenced by various factors, including the intrauterine environment, placental function, and germ cell epigenetics. While firmly establishing germ cell-dependent epigenetic inheritance mechanisms presents difficulties, this section aims to provide insights into the current understanding of the roles of epigenetic marks in male and female germ cells.

In mammals, the predominant site for DNA methylation is observed on cytosines that are positioned before a guanine, referred to as CpG sites. This process involves the methylation of the 5’ carbon atom within the cytosine molecule, facilitated by enzymes known as DNA methyltransferases (Dnmts). CpG islands represent extended DNA sequences, typically around 1000 base pairs in length, characterized by a higher density of CpG dinucleotides compared to the rest of the genome. Remarkably, approximately 50% of CpG islands encompass well-established transcription start sites. The methylation of CpG islands plays a pivotal role in the stable silencing of gene expression. However, it is important to note that the majority of CpG islands remain unmethylated (73). The analysis of DNA methylation primarily finds their focus in population-based investigations. This preference arises from the relative stability and convenience associated with high-throughput, array-based assays, making DNA methylation a prominent subject of research in understanding epigenetic mechanisms.

Histones are pivotal proteins that play a crucial role in organizing the chromatin. Post-translationally, histones undergo modifications that exert a profound influence on the compaction state of chromatin, consequently influencing gene expression. This intricate regulation gives rise to two distinctive chromatin states: euchromatin, characterized by a more relaxed and open structure, facilitating high transcriptional activity; and heterochromatin, characterized by a tightly compacted configuration that renders it transcriptionally silent.

Numerous histone modifications contribute to the dynamic orchestration of chromatin structure and gene regulation, encompassing processes such as methylation, acetylation, phosphorylation, and ubiquitination (74). These modifications constitute fundamental components of the epigenetic machinery, finely tuning gene expression patterns.

Regulatory non-coding RNAs can mainly be divided into two categories based on size: short-chain non-coding RNAs (including siRNAs, miRNAs, and piRNAs) and long non-coding RNA (lncRNAs) (75).

## Paternal inheritance

Fathers play a crucial role in the genetic inheritance of their offspring, contributing approximately half of the nuclear DNA. This inheritance not only affects the genetic predisposition to diseases but also encompasses the transmission of genetic information from the paternal side. At specific loci known as paternally derived imprinted loci, the genetic information inherited by offspring is solely from the paternal allele. This exclusivity arises because DNA methylation effectively silences the maternal allele, allowing only the paternal allele to be expressed (76).

Nonetheless, there exists compelling evidence demonstrating that factors such as paternal age and environmental exposures possess the capacity to exert a direct influence on the genetic makeup of offspring, consequently shaping their subsequent traits. This influence arises from the induction of DNA damage and the generation of *de novo* genetic mutations within the male germline (77–79). Another viable hypothesis is that the paternal environment may play a role in the selection of specific haploid genomes. This could occur through mechanisms such as altering the genotype distribution within the ejaculate or inducing mutations that subsequently impact sperm function and the probability of reproductive success (80). For instance, studies in both humans and rodents showed that male obesity impairs sperm count, motility and morphology (77, 81–85). While these genetic defects may contribute to the paternal influence on offspring phenotype, it is important to clarify that paternal transgenerational obesity is defined as alterations that manifest independently of any variations in the offspring’s genotype. This implies that changes in offspring phenotype are not contingent upon variations in their genetic makeup. This distinction makes clinical and epidemiological data interpretation difficult (86–89). In mice, however, studies have clearly demonstrated that paternal high-fat diet (HFD) feeding has adverse consequences that manifest in the subsequent generation independently from the genotype (90–95). Paternal obesity in rodents has been linked to adverse effects on offspring health, including lower mitochondrial activity, smaller offspring size, altered carbohydrate metabolism, delayed cell cycle progression, decreased blastocyst number, and decreased blastulation rate (89–92). Fullston et al. have reported that paternal effects exhibit sex-specific transmission patterns to the next generation, affecting both first-generations of paternal and maternal lines (90). Intriguingly, even when both generations of offspring were maintained on a standard diet, the authors found that F1 males pass on a predisposition to obesity and insulin resistance to their F2 female offspring. Conversely, F1 females transmit a propensity for obesity, impaired metabolic health, and insulin resistance to their F2 male descendants. It is worth noting that these intergenerational effects originating from grand-paternal HFD exposure are most pronounced in F2 male offspring born to F1 females (90). Interestingly, male neonatal overnutrition has been shown to be sufficient to promote obesity, glucose intolerance and insulin resistance later in life. Most of the metabolic disorders excluding obesity were found to be inherited to at least the two next generations through the male lineage (96). Nevertheless, all the studies that examined paternal inheritance of metabolic disorders in successive generations found that those faded in severity with each generation (90, 96, 97). Finally, although *in-utero* exposure has a stronger transgenerational transmissibility, paternal HFD exposure has been found to have an additive metabolic effect for two generations (97).

All the studies to date overwhelmingly support the idea that paternal transmission is influenced by the inheritance of epigenetic modifications transmitted across successive generations. Remarkably, both human and rodent studies have consistently revealed a robust association between dietary habits and the epigenetic alterations present in sperm (93, 97–102). For example, paternal low protein diet (LPD) feeding has been shown to promote sperm hypomethylation leading to increased adiposity, glucose intolerance, altered gut bacteria profile, and hepatic liver function resembling fatty liver disease in offspring (103). These epigenetic modifications were detected in crucial metabolic genes, including adiponectin and leptin, as well as imprinted genes such as Igf2, Peg3, Cdkn1c, and Gnas (97, 98, 104–106). Pepin et al. found that obesity induced alterations in sperm H3K4me3 profile and suggested it as a metabolic sensor of paternal obesity and the inheritance of metabolic dysfunction (107). Further pathway analysis of genes with altered H3K4me3 modification revealed an enrichment of metabolic, inflammatory, and developmental processes (107). These processes were found to correlate with offspring metabolic dysfunction and corresponded to genes enriched for H3K4me3 in embryos, which also overlapped with embryonic and placental gene expression profiles (107). In rats, paternal obesity was found to downregulate histone marks H3K4me3, H3K9me3, and H4ac, while upregulating H3K27me3 and H3ac were in placentas derived from obese male rats.

Finally, the DNA methylation pattern of SETD2 a histone methyltransferase, which methylates H3K36me2 to generate H3K36me3, has been shown to be altered in paternal HFD sperm suggesting a potential role of SETD2 as an epigenetic carrier for paternal intergenerational and transgenerational inheritance (93). It is likely that dietary habits alter multiple epigenetic modifiers’ expression, activity and function which in turn synergically promote developmental programming and it transmitted across successive generations.

## Maternal inheritance

While both parents contribute equally to the genetic makeup of offspring, it is important to consider mothers separately in the context of genetic predispositions to diseases. Mothers have the unique ability to influence offspring phenotype during gestation and lactation, making this a critical window during which maternal factors can significantly impact offspring metabolic outcomes (108–110). Notably, maternal BMI has emerged as a significant determinant of offspring health, with some studies suggesting a stronger influence than paternal BMI (25, 28, 29). However, other studies using the Northern Finland Birth Cohort found a greater effect of paternal obesity, suggesting that the mechanisms and extents of their influences may differ, underscoring the need to consider both parental roles in obesity-related research (30, 31). Nerveless, the effect of maternal obesity is rooted in developmental programming by maternal obesity, although the precise mechanisms driving this transgenerational phenomenon remain poorly defined, with existing studies proposing varying mechanisms (111, 112). Research underscores the pivotal role of nutrient availability in the uterus in determining offspring obesity risk. Both fetal undernutrition and overnutrition have been implicated in shaping altered metabolic phenotypes, indicating the involvement of multiple mechanisms in the interaction between maternal nutrition and the transgenerationally transmitted phenotype (113, 114). Understanding these intricate pathways is crucial for unraveling the complexities of maternal influence on offspring health and developing effective interventions to mitigate the risk of obesity across generations.

### Undernutrition as a driver of maternal inheritance of obesity

The thrifty phenotype hypothesis, proposed by James Neel, offers an explanation for the observed associations between poor fetal nutrition and the subsequent development of metabolic disorders (115). According to this hypothesis, individuals with a thrifty phenotype are predisposed to store energy as body fat to aid survival during times of famine or food scarcity. However, in environments abundant with nutrition, this adaptive trait can increase the risk of obesity and associated metabolic syndromes. Supporting evidence for this hypothesis comes from studies of the Dutch hunger winter, which found that individuals born during the famine were more prone to developing obesity, diabetes, and other metabolic diseases compared to those born before the famine, with a more pronounced risk observed in males (116). Furthermore, infants exposed to famine during their mother’s first trimester faced a higher risk of metabolic diseases, underscoring the heightened susceptibility of the early developmental stage to environmental influences.

In the Dutch hunger winter study, Heijmans et al. (117) identified insulin-like growth factor II (IGF2) methylation as a potential epigenetic marker distinguishing individuals exposed to famine *in utero*. IGF2, which is maternally imprinted, exhibits relatively stable methylation patterns up to middle age, enabling the detection of *in utero* epigenetic changes later in life. Their findings indicated that individuals exposed to famine early in gestation displayed lower levels of IGF2 methylation compared to those unexposed, highlighting the lasting impact of temporary environmental influences on epigenetic modifications, which may contribute to adult disease risk. Hypomethylation of Igf2 in cord blood has also been associated with an increased risk of early childhood obesity (118, 119). The imprinted gene Igf2 plays a crucial role in fetal metabolism by regulating nutrient transport to the fetus and inducing insulin resistance in the mother. Fetuses lacking placental Igf2 were found to be growth-restricted and hypoglycemic, which may further elucidate the hypomethylation of Igf2 observed in famine conditions (64).

Recent metabolic studies have revealed that individuals with a thrifty metabolism exhibit decreased activation of brown adipose tissue (BAT) in response to cold exposure, potentially contributing to their increased susceptibility to weight gain (120). This observation is supported by a recent investigation linking sperm associated antigen 7 (SPAG7) deficiency to intrauterine growth restriction, which subsequently manifests as reduced energy expenditure, obesity and insulin resistance in adulthood (69). The authors found that SPAG7-deficient mice were born underweight but developed obesity later in life and identified reduced energy expenditure as a key driver for the onset of obesity and metabolic syndrome in these mice (69). Although the underlying mechanisms remain to be fully elucidated, these studies collectively support the concept of a ‘thrifty’ phenotype, suggesting the existence of genetic or epigenetic factors predisposing offspring to increased risks of metabolic diseases.

### Overnutrition as a driver of maternal inheritance of obesity

Maternal obesity has also been recognized as a major contributor to offspring obesity, with extensive epidemiological evidence linking pre-pregnancy BMI to increased offspring weight and subsequent cardiometabolic complications in adulthood (109, 121–130). Notably, offspring born after maternal bariatric gastrointestinal bypass surgery (AMS) exhibit lower birth weights and reduced obesity compared to siblings born before maternal surgery (131, 132). Moreover, alterations in maternal hormones, including resistin, insulin-like growth factor binding protein-1 (IGFBP-1), adiponectin, visfatin, and kisspeptin-1, have been correlated with variations in offspring birth weight (133–136). However, despite the correlations observed in these studies, deciphering the causal mechanisms driving maternal transgenerational obesity inheritance remains a formidable challenge, primarily due to constraints in accessing human specimens and acquiring comprehensive data from maternal-offspring cohorts.

One hypothesis suggests the transgenerational inheritance of epigenetic modifications from obese mothers. Early studies have identified correlations between retinoid X receptor alpha (RXRA) promoter methylation and later childhood adiposity across independent cohorts (137). Leveraging advancements in Targeted Bisulfite Sequencing technology, recent studies have conducted comprehensive genome-wide analyses of fetal umbilical cord blood from offspring of obese mothers. This scrutiny unveiled a notable reduction in methylated cytosines within both CpG islands and promoter regions compared to control groups. Intriguingly, these epigenetic alterations were found to be enriched in genes associated with pathways linked to an elevated susceptibility to metabolic disorders, cancer, and cardiomyopathy (138). Replication of these findings in sibling-offspring cohorts born BMS and AMS suggests potential reversibility through surgery-induced epigenetic reprogramming (132, 139). Further investigations in sibling offspring cohorts have revealed that maternal surgical intervention induced alterations in DNA methylation and transcriptional profiles of genes implicated in insulin and leptin signaling, as well as pro-inflammatory genes. These findings underscore the capacity for maternal metabolic health improvements to modulate offspring epigenetic profiles (132).

However, deciphering the mechanisms underlying maternal obesity inheritance in human settings remains challenging. Consequently, studies using established obesity models in controlled environments have been pivotal in advancing our understanding. For instance, in-depth investigations using HFD in C57BL/6 mice for three consecutive generations have revealed a progressive exacerbation of obesity across generations, with the severity increasing from F0 to F2 (140). Moreover, the F2 generation exhibited severe glucose intolerance and insulin resistance, accompanied by increased hepatic steatosis and elevated serum levels of triglycerides, insulin, and leptin (140). These metabolic alterations were concomitant with a gradual increase in hepatic lipogenesis and endoplasmic reticulum stress genes across generations. Interestingly, the F2 generation displayed a significant reduction in accumulation of methylated histones in LXRα and ERO1-α gene promoters (140). Nevertheless, elucidating the precise contributions of maternal versus paternal transmission of obesity remains challenging due to the intricate experimental designs employed in these studies. Using a targeted experimental approach to examine the specific repercussions of maternal obesity, Huang et al. unveiled compelling insights. Their study revealed that maternal HFD not only promoted glucose intolerance and insulin resistance in the F1 generation but also led to a diminution in embryonic developmental potential. Furthermore, HFD was associated with elevated levels of reactive oxygen species (ROS) and γH2AX, coupled with a decline in mitochondrial membrane potential (MMP) within oocytes, thus instigating significant oxidative stress and DNA damage (141). Additionally, the investigation identified an elevation in Rap1-interacting factor 1 (RIF1) levels in the oocytes of HFD-fed females associated with aberrations in DNA methylation and histone modification patterns during zygotic genome activation in obese mice (141). Furthermore, RIF1 knockdown experiments using Trim-Away methods revealed that degradation of RIF1 altered the enrichment of H3K4me3 and H3K9me3, subsequently triggering the transcriptional activation of the zygotic genome activation marker Murine Endogenous Retrovirus-Leucine (MuERV-L). In a recent investigation into fetal BAT from obese females, it was found that maternal obesity triggers an increase in the expression of Dio3, encoding deiodinase 3 (D3), thereby leading to the catabolization of triiodothyronine (T3). Simultaneously, the authors uncovered a suppression of the maternally imprinted long noncoding RNA, Dio3 antisense RNA (Dio3os), resulting in intracellular T3 deficiency and subsequent inhibition of BAT development. Furthermore, the investigators noted a higher degree of methylation in the Dio3os promoter region in the oocytes of obese mothers, a modification that persisted in the offspring (142). Conversely, Wang et al. demonstrated that maternal obesity suppressed genes associated with myogenesis and brown adipogenesis while promoting white adipogenesis during fetal BAT development by enhancing miR-204-5p expression, consequently leading to the suppression of PGC1α and Sirt1 (143). Collectively, these findings suggest a complex interplay of epigenetic modifications underlying the inheritance of maternal obesity, potentially persisting across generations.

It is noteworthy that sexual dimorphism in transgenerational inheritance of obesity has been reported, indicating a more complex mechanism than initially presumed. For instance, oocytes exposed to obesity have been previously found to accumulate and transmit dysfunctional mitochondria to offspring due to an impaired ability to activate mitophagy (144). Subsequent studies across generation have demonstrated the transmission of dysfunctional mitochondria to the second and third generations through the female germline (145). Furthermore, recent research has shown that maternal obesity induced by a HFD disrupts genomic methylation in oocytes, with at least some of the altered methylation transmitted to F2 oocytes and livers via females. Interestingly, the involvement of melatonin in regulating the hyper-methylation of HFD oocytes has been identified, with melatonin increasing the expression of DNMT3a and DNMT1 mediated by the cAMP/PKA/CREB pathway (146). These findings highlight the importance of considering significant distinctions in the mechanisms of maternal inheritance of obesity between female and male offspring in future studies.

### Fetal nutritional availability as a driver of maternal inheritance of obesity

The complex interplay occurring at the maternal-fetal interface exerts a profound influence on long-term fetal health outcomes, underscoring the need to delve into placental genomic regulations and nutrient sensing pathways as potential contributors to disrupted metabolic phenotypes in offspring (147). HFD consumption before and during pregnancy has been found to enhance nutrient transport and fetal overgrowth in both human and murine studies (148–151). These findings have been linked to the upregulated expression of the mammalian target of rapamycin (mTOR) complex 1, which modulates nutrient transporter expression. Interestingly, vasoactive intestinal peptide (VIP) has been identified as a regulator of glucose and amino acid uptake, exerting its effects by increasing GLUT1 and mTOR gene expression (152). Notably, offspring of VIP-deficient mothers exhibit a marked reduction in body weight (153). Maternal inheritance of obesity has also been associated with dysregulation of circulating steroid hormones during pregnancy. In addition to steroid hormone dysregulation, maternal obesity is associated with alterations in a wide range of hormones, growth factors, and cytokines, which can significantly affect pregnancy outcomes. Page L et al. provide an extensive review of these dysregulations, highlighting the complex endocrine environment in obese pregnancies and its potential implications for both maternal and fetal health (154). One study in rodents demonstrated that maternal obesity resulted in low fetal weight in the F1 generation, accompanied by modified DNA methylation and altered expression of the nuclear hormone receptor RXRα in a sex-dependent manner (155). Methylation of RXRα has also been linked to childhood adiposity (137). Examination of placentas from obese women reveals diminished mitochondrial β-oxidation of fatty acids (FA) and lipid accumulation in late pregnancy, fostering a lipotoxic environment (156). The authors found a significant reduction in genes associated with FA oxidation, uptake, synthesis, and storage, with pronounced effects notably observed in placentas of male fetuses (156).. Furthermore, alterations in placental genes implicated in modulating offspring glucose and insulin metabolism under the stress of maternal obesogenic conditions have been documented (152). Taken together, these findings suggest that maternal obesity-induced alterations in nutritional uptake may constitute critical determinants of maternal inheritance of obesity. Notably, evidence suggests that the action of epigenetic modifiers is sensitive to changes in dietary components and cellular metabolism intermediates, linking nutrition and energy metabolism to gene expression plasticity (157).

## Environmental influence

Considering the inheritability and genetic basis of obesity, it’s crucial to also examine gene-environment interactions as potential contributors to the mechanism of inheritance Studies have implicated exposure to environmental toxicants in the transgenerational inheritance of obesity and the development of DNA methylation sperm epimutations. Notable among these chemicals are plastics such as BPA, phthalates DEHP and DBP, bisphenol S, the herbicide glyphosate, insecticides like DDT and methoxychlor, the biocide tributyltin, the combustion byproduct benzo[a]pyrene, and hydrocarbons from jet fuel (158–165). Most of these chemicals are classified as endocrine disruptors and have been labeled as “obesogens,” capable of promoting obesity by increasing fat cell count, fat storage, and affecting appetite mechanisms. For instance, exposure to the estrogenic endocrine disruptor BPA has been linked to deregulated genomic methylation and hydroxymethylation (166, 167). Prenatal exposure to BPA in humans has been associated with early childhood obesity and methylation changes at the mesoderm-specific transcript homologue (MEST) locus (168). Studies on BPA-treated mouse spermatogonia showed reduced expression of Dnmt1, while exposure during oocyte maturation altered histone modifications due to oxidative stress (169, 170). BPA exposure has also been found to affect TET enzyme expression and function, leading to altered levels of 5hmC at several imprinted loci (167). This suggests a potential mechanism through which environmental toxicants can disrupt long-term imprinted gene regulation, ultimately contributing to obesity.

Moreover, some studies suggest that the effects of obesogens can be inherited across multiple generation. A transgenerational mouse study showed that grandparental exposure to tributyltin (TBT) resulted in increased fat depots in offspring extending to the F3 generation (171). Additionally, environmental factors such as paternal cold exposure have been implicated in influencing offspring obesity. Human studies have shown that individuals conceived in colder months exhibit higher BAT activation. Corresponding mouse studies demonstrated that paternal cold exposure affected sperm epigenetic programming and enhanced BAT activity in offspring (172).

Another environmental factor of interest is the maternal gut microbiome. Maternal diet influences the maternal gastrointestinal tract (GIT) microbiota, vaginal microbiota, and breast milk composition, which in turn influence the colonization of the fetus’ GIT either *in utero* or postnatally. The fetal microbiome plays a crucial role in GIT mucosa development and may be linked to obesity (173). Vaginal delivery is considered vital for infants to acquire bacterial communities resembling their mothers’, while cesarean delivery disrupts this process, leading to changes in offspring immune and metabolic programming (174). Indeed, studies have reported that infants born via cesarean delivery are more predisposed to obesity (175).

## Current treatments and future direction

Treatments plans ranging from as simple as regulating the diet to as complex as surgery are available for the management of obesity. However, few methods are currently recommended for obese women during pregnancy or in the early stages of offspring development. Nonpharmacological interventions include dietary adjustments and increased physical activity, both of which been shown to confer benefits mediated through epigenetic mechanisms (176, 177). Notably, exercise during pregnancy has been shown to prevent the reduction in placental vascularization and fetal overgrowth associated with maternal obesity (178). Mechanistically, maternal exercise was found to downregulated mTOR protein expression mTOR and amino acid transporters promoting a healthier fetal outcome (179). Furthermore, maternal exercise during pregnancy as also found to improve fetal metabolic health through a vitamin D receptor-mediated increase in placental of superoxide dismutase 3 (SOD3), which in turn enhances liver function, and improves glucose tolerance in offspring (180). Extensive reviews have highlighted the effects of maternal and paternal exercise on offspring metabolism (181–184), underscoring the potential of regular exercise to break the cycle of increased metabolic risk across generations. However, the translation of these findings to the human population remains to be addressed.

Pharmacological treatment options typically target patients with a BMI ranging from 27 to 30 kg/m², with discontinuation recommended if less than 5% of the target weight is lost within three months of starting the medication (185). Currently, there are seven FDA-approved drugs for long-term weight loss, including Semaglutide, Setmelanotide, Gelesis100, Liraglutide, Bupropion-naltrexone, Phentermine-topiramate, and Orlistat (**Table 2**) (185). Of these, semaglutide and liraglutide, which are glucagon-like peptide 1 (GLP-1) agonists, show the most promising results, with semaglutide demonstrating the highest placebo-subtracted weight loss percentage (186, 187). Off-label drugs for weight loss include Bupropion, Metformin, Pramlintide, Sodium glucose cotransporter 2 (SGLT-2) inhibitors, Topiramate, and Zonisamide (185). A summary of the most common FDA-approved drugs for long-term weight loss, along with those currently in clinical trials, is provided in **Table 2**. However, none of these drugs have been tested in the context of maternal obesity and its impact on future offspring.

**Table 2:** List of drugs approved or currently in trial for obesity treatment and their targets for treatment (185).

Drug	Status	Mechanism/Target
Tirzepatide	FDA Approved (2023)	Dual agonist (GIP, GLP-1)
Semaglutide	FDA Approved (2021)	GLP-1 agonist
Setmelanotide	FDA Approved (2020)	MC4R agonist (for monogenic obesity)
Gelesis100	FDA Approved (2019)	Oral hydrogel that expands in the stomach, creating satiety
Liraglutide	FDA Approved (2014)	GLP-1 agonist
Bupropion-naltrexone	FDA Approved (2014)	**Bupropion**: Dopamine and norepinephrine re-uptake inhibitor **Naltrexone**: Opioid receptor antagonist
Phentermine-topiramate	FDA Approved (2012)	**Phentermine**: Sympathomimetic (appetite-suppressing) **Topiramate**: Antiepileptic
Orlistat	FDA Approved (1999)	Gastric/Pancreatic lipase inhibitor
Diethylpropion	FDA Approved (1979)	Sympathomimetic
Phentermine	FDA Approved (1959)	Sympathomimetic
Metformin	Off-Label	Antihyperglycemic agent
Pramlintide	Off-Label	Mimics pancreatic hormone amylin which regulates post-prandial glucose
Canagliflozin, Dapagliflozin, Ertugliflozin, and Empagliflozin	Off-Label	SGLT-2(Sodium glucose cotransporter 2) inhibitors
Zonisamide	Off-Label	Antiepileptic
Orforglipron	Phase III Clinical Trials	Oral GLP-1 agonist
Retatrutide	Phase III Clinical Trials	Triple-hormone-receptor agonist (GIP, GLP-1, GCG)
Mazdutide	Phase III Clinical Trials	Dual agonist (GLP-1, GCG)
Pemvidutide	Phase II Clinical Trials	Dual agonist (GLP-1, GCG)
Danuglipron	Phase II Clinical Trials	GLP-1 agonist
S-309309	Phase II Clinical Trials	MGAT2(monoacylglycerol acyltransferase-2) inhibitor
ARD-101	Phase II Clinical Trials	TAS2R(bitter taste receptor) agonist
APH-012	Phase II Clinical Trials	Restores intestinal hormone response
Bimagrumab	Phase II Clinical Trials	ACTRII(activin type II receptor) inhibitor

## Conclusion

**Figure 3:**
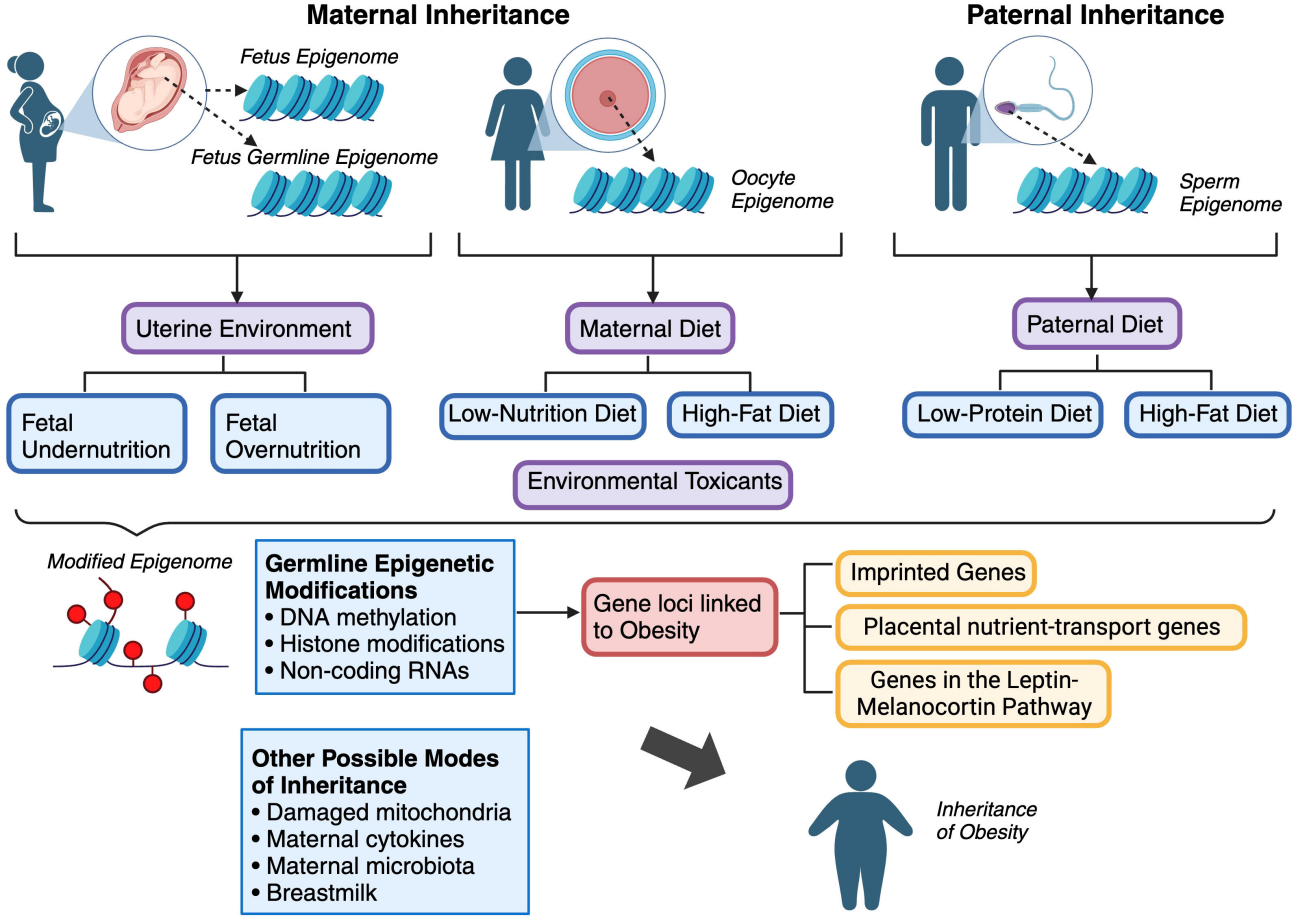
Summary of the various modes of inheritance of obesity.

Current research unequivocally illustrates the transgenerational transmission of obesity (**Figure 3**). Recent studies in both mice and humans have revealed that both undernutrition and overnutrition contribute to metabolic disorders in offspring, shedding light on some of the underlying mechanisms. However, numerous critical areas warrant future investigation to comprehensively grasp the central mechanisms that drive the perpetuation of obesity across generations. With emerging evidence indicating sexual dimorphism in the transmission of obesity, elucidating its extent will be invaluable for tailoring future pharmacological interventions. Additionally, further studies are imperative to thoroughly dissect the impact of maternal obesity on the metabolic organs of offspring and to ascertain whether the pathophysiological mechanisms promoting cardiometabolic complications in adulthood are equivalent across generations of obesity. For instance, because obesity occurs earlier and manifests more severely in offspring, future research should adopt a holistic approach to analyze inherited epigenetic alterations in the metabolic organs of offspring. The placental–fetal system represents another focal point for offspring developmental programming, given its significant role during the critical windows of prenatal development. Identifying the mediating factors and signaling pathways is essential for human translation, particularly given the escalating global obesity epidemic, which renders these issues increasingly pertinent for the future.

## Author contributions

SS: Writing – original draft. DL: Writing – original draft. NR: Conceptualization, Funding acquisition, Project administration, Resources, Supervision, Writing – review & editing.
